# Major Grass Pollen Allergen Components and Cross-Reactive Carbohydrate Determinants in Mugwort-Sensitized Child Patients With Allergic Respiratory Disease in Western China

**DOI:** 10.3389/fped.2022.816354

**Published:** 2022-04-14

**Authors:** Chenxi Liao, Xiangqing Hou, Liting Wu, Wenting Luo, Hong Zhang, Xin Sun, Yongmei Yu, Xiaohua Douglas Zhang, Baoqing Sun

**Affiliations:** ^1^State Key Laboratory of Respiratory Disease, Department of Allergy and Clinical Immunology, National Clinical Research Center of Respiratory Disease, Guangzhou Institute of Respiratory Health, First Affiliated Hospital of Guangzhou Medical University, Guangzhou Medical University, Guangzhou, China; ^2^Department of Clinical Laboratory, The People's Hospital of Zhuhai, Zhuhai, China; ^3^Faculty of Health Sciences, University of Macau, Macao, China; ^4^Department of Pediatrics, Gansu Provincial Hospital, Lanzhou, China; ^5^Department of Pediatrics, Xijing Hospital, The Fourth Military Medical University, Xi'an, China; ^6^Department of Pediatrics, First Affiliated Hospital of Kunming Medical University, Kunming, China

**Keywords:** component-resolved diagnosis (CRD), cross-reactive carbohydrate determinants (CCD), pollen allergen component, specific IgE (sIgE), EUROBlotMaster

## Abstract

Mugwort is a common pollen allergen in western China, and this study aimed to investigate the patterns of molecular sensitization to major grass pollen allergens (mugwort, ragweed, bermuda grass, and timothy grass) and cross-reactive carbohydrate determinants (CCD) in children who were sensitized to mugwort in western China. Serum-specific IgE (sIgE) of major allergen components and CCD were detected among 121 mugwort SPT-positive children *via* the EUROBlotMaster system if the mugwort-sIgE was positive (MSP). A CCD inhibition test was further performed on the serum of patients with positive CCD-sIgE. Latent class analysis was used to identify the patterns of potential sensitization to major grass pollen allergens. Of a total of 100 patients with mugwort-sIgE positive (MSP), 52.0, 41.0, and 31.0% of them were positive to Art v 1, Art v 3, and Art v 4, respectively. An optimal model with three latent classes was determined using grass pollen allergens, components, and CCD. The sensitization patterns can be summarized as (1) MSP and cosensitized to ragweed, bermuda grass, and timothy grass (23.74%); (2) MSP and cosensitized to Art v 1 (54.08%); (3) MSP and cosensitized to Art v 4, Cyn d 12, Phl p 12 (22.18%). Additionally, CCD sIgE levels had a significant positive correlation with ragweed, bermuda grass, and timothy grass (*P* < 0.05), and CCD-Inhibitor can highly inhibit the above allergens sIgE. Our findings suggest that Art v 4 was the typical cross-reaction component of mugwort, which is cosensitized to Phl p 12 and Cyn d 12. A wide cross-reaction among ragweed, bermuda grass, and timothy grass caused by CCD was observed.

## Introduction

It is reported that the incidence of many allergic diseases, such as allergic rhinitis and asthma, is widely increased worldwide in the past decades, especially in children (1, 2). A multicenter study was performed in China to show that the prevalence rate of asthma in most urban children increased by about 0.5 and 1.5 times from 1990 or 2000 to 2010 years, respectively (3), which poses a substantial health burden to children.

In the late summer or autumn, mugwort releases a large amount of pollen, which can not only float in the air, but also cause cough, wheezing, runny nose, and other allergic symptoms and, therefore, brings great trouble to patients with allergic diseases. Mugwort (Artemis vulgaris) is widely distributed in Europe, North America, and some parts of Asia. Previous studies report that the sensitization rate of mugwort in Italy is 17% (4) and even can reach 44.3% in Hungary (5). Furthermore, in Germany and Switzerland, mugwort is also one of the important causes of allergy (6–8). A multicenter study conducted in northern China showed that about 24% of subjects in patients with asthma and/or rhinitis were sensitized to a skin prick test (SPT) for mugwort (9). The results of the serum allergen chip of 100 allergy patients from China showed that mugwort-sIgE had the highest reactivity among pollen allergens (10). Additionally, the positive rate of sIgE of mugwort can reach 88% in 50 serum samples from patients with allergic rhinitis caused by grass pollen from Beijing, China (11).

Concurrent sensitization of multiple allergens is a natural phenomenon (12). For instance, it is very common that ragweed, bermuda grass, timothy grass, and mugwort are simultaneously positive among allergic patients (13–15). The effective identification of molecular sensitization patterns among allergic children would be beneficial for guild diagnosis and therapy. Although component-resolved diagnosis (CRD) has been introduced into the allergy discipline in many studies, most of them focus on the components of a specific allergen, and to our best knowledge, few studies investigate the components of allergens in different pollens. A large-scale epidemiology investigation reveals that the prevalence patterns of allergen sensitization in China are highly complicated, which results in regional differences in the distribution of allergens (16). The western region of China is characterized by long latitude and longitude, distributed in different temperature zones with rich vegetation types and numerous characteristic pollen species. Meanwhile, the economic conditions in western China are relatively backward; thus, there is no well-conducted research to comprehensively explore the associations between pollens and allergy in this area.

CRD technology was utilized to detect the serum sIgE of mugwort, bermuda grass, timothy grass, and ragweed allergen components and cross-reactive carbohydrate determinants (CCD) simultaneously, which aimed to obtain the pollen allergen component map and to investigate the associations between different pollen allergen components and CCD-sIgE. In addition, the diagnostic indexes of pollen allergen specificity were determined by CCD inhibition test.

## Materials and Methods

### Study Design

This was a cross-sectional study, and the samples originated from four medical centers in the western region of China, including Lanzhou, Yinchuan, Xi'an, and Kunming. Child patients with mugwort sensitization from the Allergy Information Repository of the State Key Laboratory of Respiratory Disease (AIR-SKLRD) (17) during January 2020 to December 2020 in western China were included by the following inclusion criteria: (1) patients with a history of pollen exposure and respiratory allergic symptoms, such as wheezing, dyspnea, sneezing, runny nose, nasal obstruction, or nasal itching; (2) SPT of mugwort was positive and sIgE level of mugwort was larger than 0.35 kU/L; (3) aged 0–15 years. Additionally, patients with specific immunotherapy, cancer, autoimmune diseases, and parasitic diseases were excluded. Last, a total of 100 child patients aged 3–15 years sensitized to mugwort underwent sIgE detection, and the test allergens, including mugwort and allergen components (Art v 1, Art v 3 and Art v 4), ragweed and allergen components (Amb a 1), bermuda grass and allergen components (Cyn d 1 and Cyn d 12), and timothy grass and allergen components (Phl p 1, Phl p 4, Phl p 5, Phl p 7, and Phl p 12), and CCD. The diagnosis of asthma and allergic rhinitis were based on the Guidelines of Global Initiative for Asthma (GINA) (18) as well as Allergic Rhinitis and its Impact on Asthma (ARIA) (19), respectively.

### sIgE

Serum samples of patients were obtained by 10-min-centrifugation at 3,000 g of venous blood collected with separation gel containing vacutainer tubes. Aliquots of serum were stored at −80°C in AIR-SKLRD. The serum sIgE levels were detected by the EUROBlotMaster system (EUROIMMUN Medizinische Labordiagnostika AG, Lübeck, SH, Germany) according to the instructions provided by the manufacturer. Art v 1 and Amb a 1 were natural components and the others were recombinant components. Levels of sIgE were expressed in kU/L, and results of sIgE class based on RAST classification were defined as follows: Class 0, <0.35 kU/L; Class 1, 0.35–0.70 kU/L; Class 2: 0.70–3.50 kU/L; Class 3: 3.50–17.50 kU/L; Class 4: 17.50–50.00 kU/L; Class 5: 50.00–100.00 kU/L and Class 6: ≥100.00 kU/L.

### CCD Inhibition Test

1:20 Anti-CCD absorbent (EUROIMMUN Medizinische Labordiagnostika AG, Lübeck, SH, Germany) was added to the samples to be tested, and then mixed well and incubated at room temperature (18–25°C) in a shaker for 1 h. Immediately afterward, sIgE of mugwort and allergen components (Art v 1, Art v 3 and Art v 4), ragweed and allergen components (Amb a 1), bermuda grass and allergen components (Cyn d 1 and Cyn d 12), and timothy grass and allergen components (Phl p 1, Phl p 4, Phl p 5, Phl p 7, and Phl p 12), and CCD were tested *via* the EUROBlotMaster system. The inhibition rates of CCD on pollen allergens were calculated by (sIgE after CCD inhibition minus the corresponding values before CCD inhibition and then divide by before levels) (20, 21).

### Statistical Analysis

Quantitative data were presented as median (first quartile, third quartile) because of their apparent skewed distribution, and the Mann–Whitney *U*-test was utilized to perform group comparison. Categorical data were reported as a frequency and percentage proportion, and the Chi-square test (χ^2^) was used to compare the variance of data among the groups. A Venn diagram was created to depict the overlap sensitization of mugwort allergen components. Spearman correlation analyses were performed to investigate the associations among different grass pollen allergens and CCD. Latent class analysis (LCA) was utilized to select optimal potential sensitization patterns using 15 grass pollen allergens or components and CCD based on the lowest BIC and interpretability.

All tests were two-sided and *P* < 0.05 was considered statistically significant. Statistical analyses were conducted with SPSS 25.0 (Chicago, IL) and SAS 9.4 software. The figures were drawn by R-studio 1.2.5001 (Copyright 2009–2018 R-studio, Inc.).

## Results

### Clinical Characteristics of Study Patients and Molecular Sensitization Patterns of Mugwort

Among the mugwort-sIgE positive patients (*n* = 100), 65% of them were boys. The median (first quartile, third quartile) age was 7 (6, 10) years. A total of 43 children were diagnosed with allergic rhinitis and 20 as asthma in this study, additionally, 37% (*n* = 37) of sIgE positive children suffered from rhinitis and asthma comorbidity. We divided all participants into preschool- (42%) and school-aged children (58%) according to the cutoff age of 6 years: 52.0% (52/100), 41.0% (41/100), and 31.0% (31/100) showed positive sIgE to Art v 1, Art v 3, and Art v 4, respectively. The details can be seen in Table 1.

**Table 1:** The characteristics of patients with mugwort sensitization.

**Variables**	**Total** ***N*** **= 100**	**Mugwort sensitized**		***P*** **-value**
		**CCD positive** ***N*** **= 16**	**CCD negative** ***N*** **= 84**	
Sex, *n* (%)				**0.360**
Male	65 (65.0)	12 (75.0)	53 (63.1)	
Female	35 (35.0)	4 (25.0)	31 (36.9)	
Age, *n* (%)				**0.208**
≤ 6 years	42 (42.0)	9 (56.3)	33 (39.3)	
6 ~ 15 years	58 (58.0)	7 (43.8)	51 (60.7)	
Diseases, *n* (%)				**0.208**
AR	43 (43.0)	8 (50.0)	35 (41.7)	
AS	20 (20.0)	5 (31.3)	15 (17.9)	
AR + AS	37 (37.0)	3 (18.8)	34 (40.5)	
Region, *n* (%)				**0.385**
Lanzhou	29 (29.0)	4 (25.0)	25 (29.8)	
Yinchuan	27 (27.0)	7 (43.8)	20 (23.8)	
Xi'an	23 (23.0)	2 (12.5)	21 (25.0)	
Kunming	21 (21.0)	3 (18.8)	18 (21.4)	
Sensitization, *n* (%)				
Art v 1	52 (52.0)	3 (18.8)	49 (58.3)	0.004
Art v 3	41 (41.0)	8 (50.0)	33 (39.3)	0.425
Art v 4	31 (31.0)	6 (37.5)	25 (29.8)	0.750
Amb a	36 (36.0)	14 (87.5)	22 (26.2)	<0.001
Amb a 1	7 (7.0)	3 (18.8)	4 (4.8)	0.140
Cyn d	42 (42.0)	16 (100.0)	26 (31.0)	<0.001
Cyn d 1	12 (12.0)	2 (12.5)	10 (11.9)	1.000
Cyn d 12	20 (20.0)	4 (25.0)	16 (19.0)	0.838
Phl p	36 (36.0)	12 (75.0)	24 (28.6)	<0.001
Phl p 1	15 (15.0)	3 (18.8)	12 (14.3)	0.939
Phl p 4	4 (4.0)	4 (25.0)	0 (0.0)	<0.001
Phl p 5	2 (2.0)	0 (0.0)	2 (2.4)	1.000
Phl p 7	2 (2.0)	1 (6.3)	1 (1.2)	0.296
Phl p 12	24 (24.0)	4 (25.0)	20 (23.8)	1.000

*AR, allergic rhinitis; AS, asthma*.

**Figure 1:**
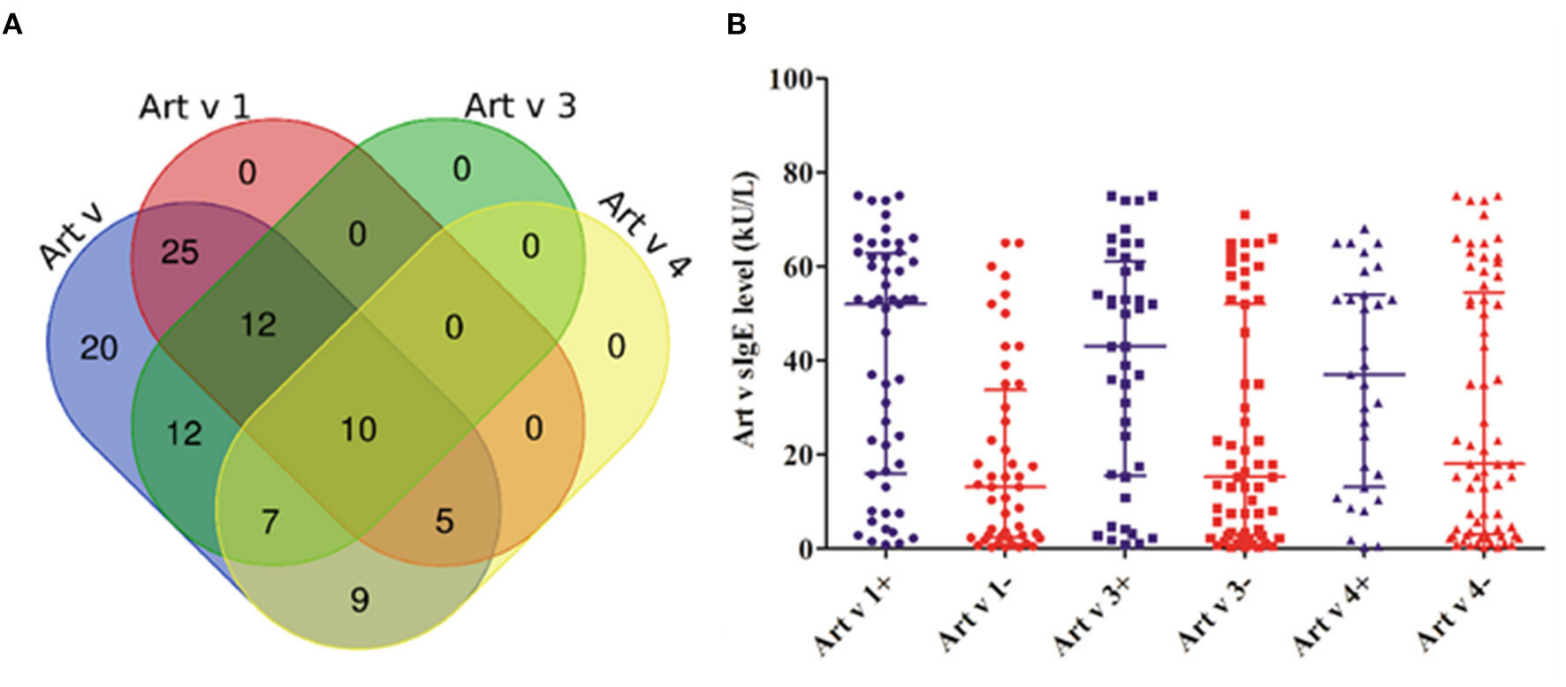
The cosensitization of mugwort and its components. **(A)** The Venn diagram shows the number of patients what were cosensitized. **(B)** The comparison of different mugwort sIgE levels between positive (+) and negative (–) patients in Art v 1, Art v 3, and Art v 4.

The cosensitization rate of the three mugwort component allergens (Art v 1, Art v 3, and Art v 4) reached 10.0% (Figure 1A). The sIgE level of mugwort in Art v 1–positive patients (52.0, 16.1–62.5 kU/L) was significantly higher than that in Art v 1–negative patients (13.1, 2.5–32.5 kU/L) (*P* < 0.001). Similarly, the sIgE level of mugwort in Art v 3–positive patients (43.0, 15.8–60.0 kU/L) was significantly higher than that in Art v 3–negative patients (15.3, 2.8–52.0 kU/L) (*P* = 0.004) (Figure 1B). However, no significant difference in sIgE level was observed between Art v 4–positive and –negative patients.

### Mugwort SIgE-Positive Patients' Cosensitization With Other Grass Pollen Allergens and Components

From Table 1, we can conclude that 36.0% (36/100) of mugwort-sensitized patients had ragweed sIgE-positive, and only 13.9% (5/36) of ragweed sIgE-positive patients showed Amb a 1–positive. Besides this, these five patients also presented Art v 3–positive. Moreover, 42.0% (42/100) of mugwort-sensitized patients had bermuda grass sIgE-positive, among which 21.4% (9/42) showed Cyn d 1–positive and 38.1% (16/42) showed Cyn d 12–positive. Moreover, these 16 Cyn d 12–positive patients were combined with Art v 4 and Phl p 12 positives. Additionally, a total of 36 patients showed timothy grass sIgE-positive, of which 44.4% (16/36) were found to be Phl p 12 positive, 27.8% (10/36) showed Phl p 1 positive, and 11.1% (4/36) showed Phl p 4 positive. It can be observed that 4 Phl p 4-positive patients were combined with Art v 3, ragweed, bermuda grass, and CCD positive. The results also suggest that only 5.6% (2/36) of participants with timothy grass sIgE-positive showed Phl p 5 and Phl p 7 positives.

### The Correlation of Different Major Grass Pollen Sensitization and CCD Testing Results

**Figure 2:**
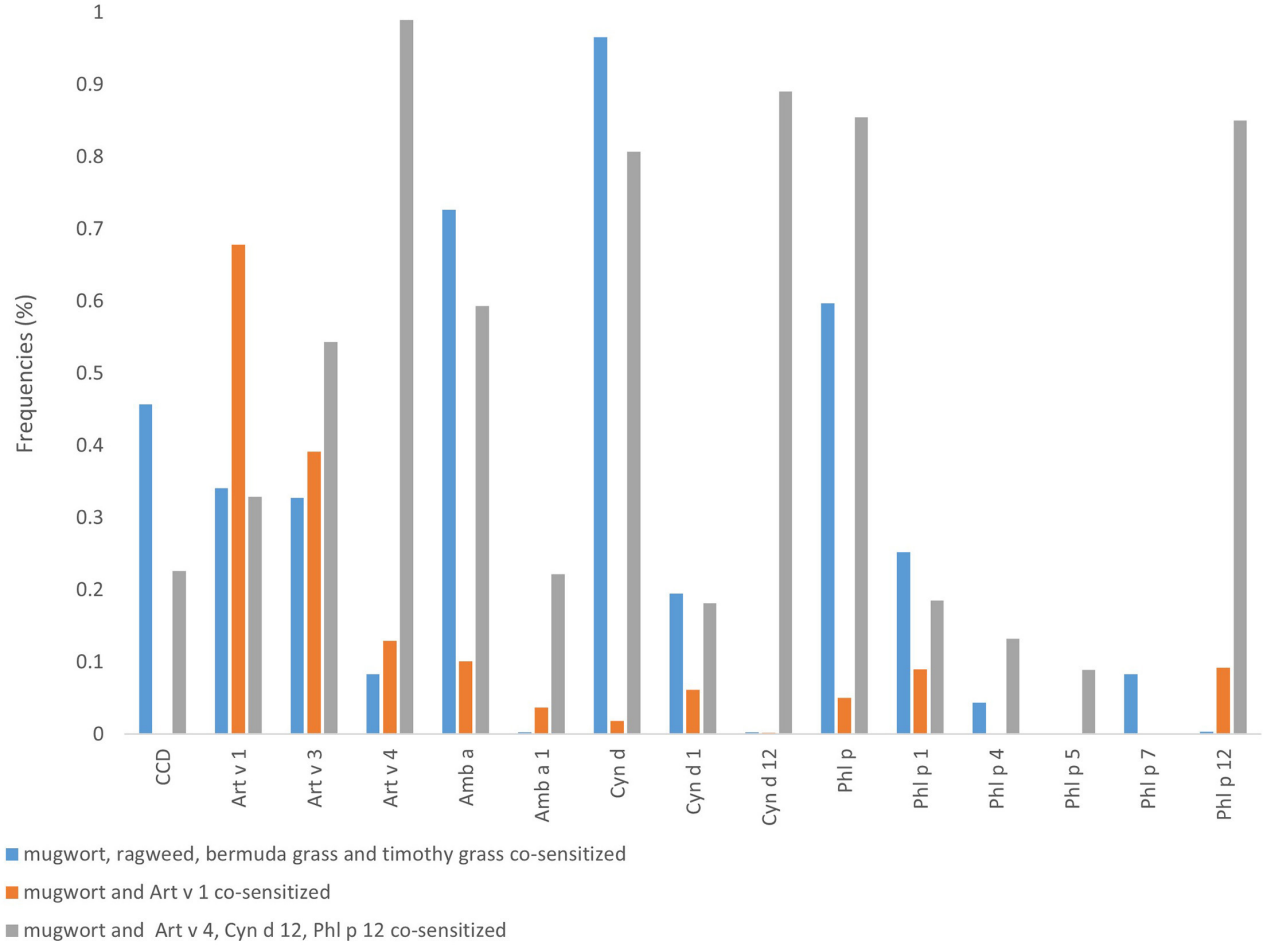
Spearman correlation analysis among various grass pollen allergens and CCD. The numbers in the figure are Spearman's correlation coefficient.

Figure 2 shows there was a moderate correlation between Art v 1 and mugwort (*r*_*s*_ = 0.62, *P* < 0.001), whereas Art v 3 and Art v 4 had a weak correlation with mugwort (*r*_*s*_ = 0.28, *P* = 0.307; *r*_*s*_ = 0.18, *P* = 0.375, respectively). Moreover, the results also suggest that there was a significant correlation between Art v 3-sIgE and Amb a 1-sIgE (*r*_*s*_ = 0.32, *P* = 0.001). It is confirmed that Cyn d 12-sIgE was highly correlated with Art v 4-sIgE (*r*_*s*_ = 0.78, *P* < 0.001) and Phl p 12-sIgE (*r*_*s*_ = 0.84, *P* < 0.001). Additionally, the results also reveal that Phl p 12-sIgE was highly correlated with Art v 4-sIgE (*r*_*s*_ = 0.88, *P* < 0.001).

16.0% (16/100) of mugwort-sensitized patients showed CCD sIgE positive in this study. The comparison of clinical characteristics and grass pollen allergen component's sIgE testing results between CCD positive patients and negative ones are described in Table 1. Compared with those who were CCD-negative, children with positive CCD results had significant higher positive rates of Amb a (87.5 vs. 26.2%), Cyn d (100.0 vs. 31.0%), Phl p (75.0 vs. 28.6%), Phl p 4 (25.0 vs. 0%) and lower of Art v 1 (18.8 vs. 58.3%). Spearman correlation analysis reveals (Figure 2) that the sIgE level of CCD was significantly negatively correlated with the sIgE level of Art v 1 (*r*_s_ = −0.3, *P* = 0.002), and significantly positively with ragweed (*r*_s_ = 0.5, *p* < 0.001), Amb a 1 (*r*_s_ = 0.23, *P* < 0.019), Bermuda (*r*_s_ = 0.56, *P* < 0.001), timothy (*r*_s_ = 0.39, *P* < 0.001), and Phl p 4 (*r*_s_ = 0.53, *P* < 0.001).

### Cosensitization Patterns of Major Grass Pollen Allergen Components and CCD

**Figure 3:**
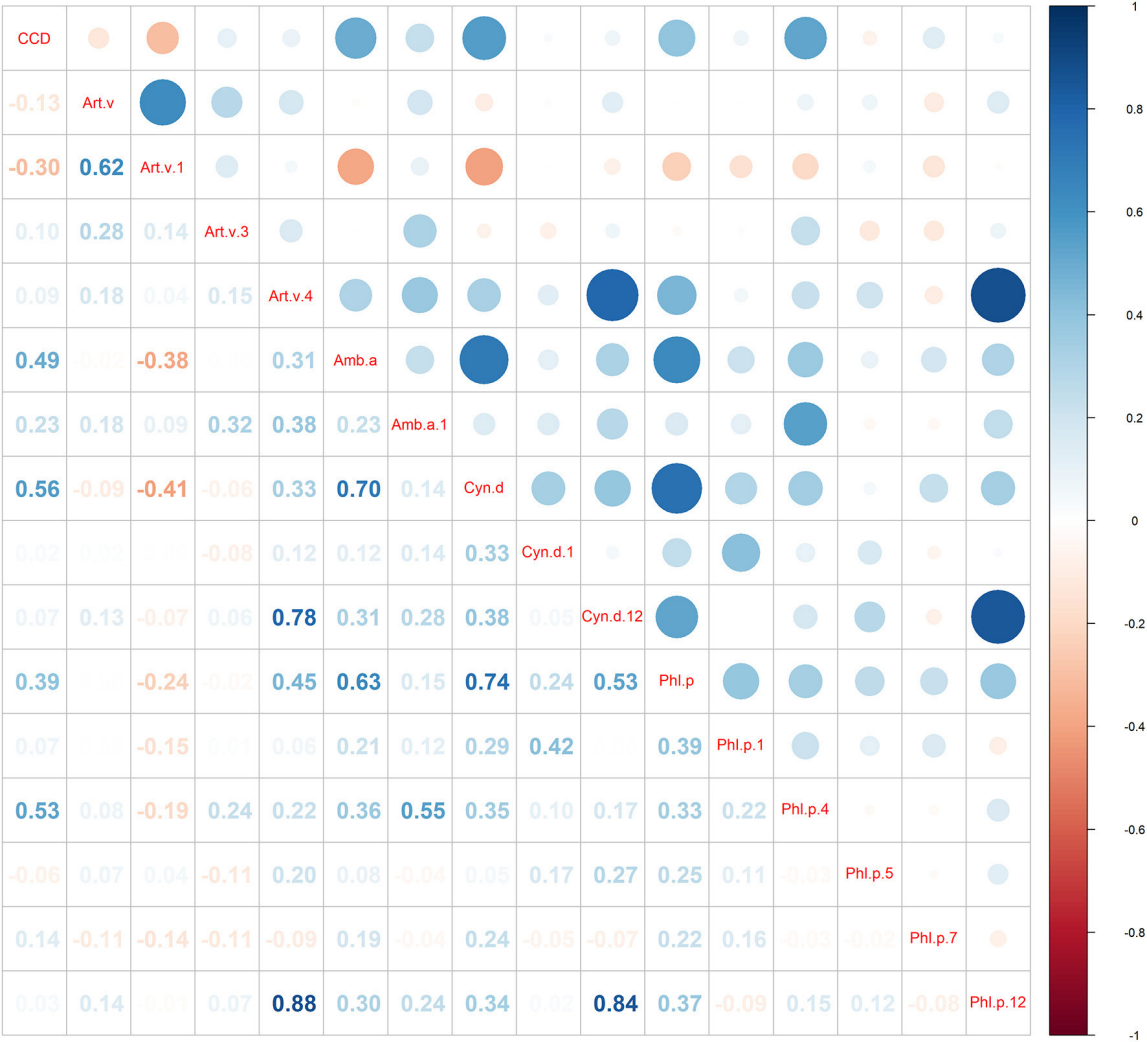
Sensitization patterns identified by latent class analysis. Class proportions shown in the figure are computed based on most likely class membership.

The results of LCA revealed that there were the lowest BIC in the model with three potential classes (Supplemental Table 1). Based on the results of conditional probabilities (Table 2, Figure 3) and allergen sensitization patterns, the three latent classes were labeled as (1) mugwort-sIgE positive and co-sensitized to ragweed, bermuda grass, timothy grass (23.74%); (2) mugwort-sIgE positive and co-sensitized to Art v 1 (54.08%); (3) mugwort-sIgE positive and co-sensitized to Art v 4, Cyn d 12, Phl p 12 (22.18%).

**Table 2:** Conditional probabilities of pollen sensitization patterns and outcomes in children who sensitized to mugwort.

**Pattern risk factors**	**Outcome**	**Class 1 (*n* = 24)**	**Class 2 (*n* = 54)**	**Class 3 (*n* = 22)**
CCD	Positive	0.4573	0.0011	0.2263
	Negative	0.5427	0.9989	0.7737
Art v 1	Positive	0.3405	0.6785	0.3287
	Negative	0.6595	0.3215	0.6713
Art v 3	Positive	0.3271	0.3916	0.5431
	Negative	0.6729	0.6084	0.4569
Art v 4	Positive	0.0828	0.1294	0.9897
	Negative	0.9172	0.8706	0.0103
Amb a	Positive	0.7266	0.1012	0.5936
	Negative	0.2734	0.8988	0.4064
Amb a 1	Positive	0.0024	0.0371	0.2219
	Negative	0.9976	0.9629	0.7781
Cyn d	Positive	0.9658	0.0184	0.8071
	Negative	0.0342	0.9816	0.1929
Cyn d 1	Positive	0.1945	0.0616	0.1816
	Negative	0.8055	0.9384	0.8184
Cyn d 12	Positive	0.0028	0.0016	0.8906
	Negative	0.9972	0.9984	0.1094
Phl p	Positive	0.5970	0.0505	0.8545
	Negative	0.4030	0.9495	0.1455
Phl p 1	Positive	0.2524	0.0902	0.1850
	Negative	0.7476	0.9098	0.8150
Phl p 4	Positive	0.0434	0.0002	0.1324
	Negative	0.9566	0.9998	0.8676
Phl p 5	Positive	0.0003	0.0001	0.0891
	Negative	0.9997	0.9999	0.9109
Phl p 7	Positive	0.0834	0.0001	0.0003
	Negative	0.9166	0.9999	0.9997
Phl p 12	Positive	0.0035	0.0923	0.8501
	Negative	0.9965	0.9077	0.1499
Latent class probabilities		0.2374	0.5408	0.2218

*(1) Class1: MSP and co-sensitized to ragweed, bermuda grass, timothy grass; (2) Class2: MSP and co-sensitized to Art v 1; (3) Class3: MSP and co-sensitized to Art v 4, Cyn d 12, Phl p 12*.

### CCD Inhibition Test

**Figure 4:**
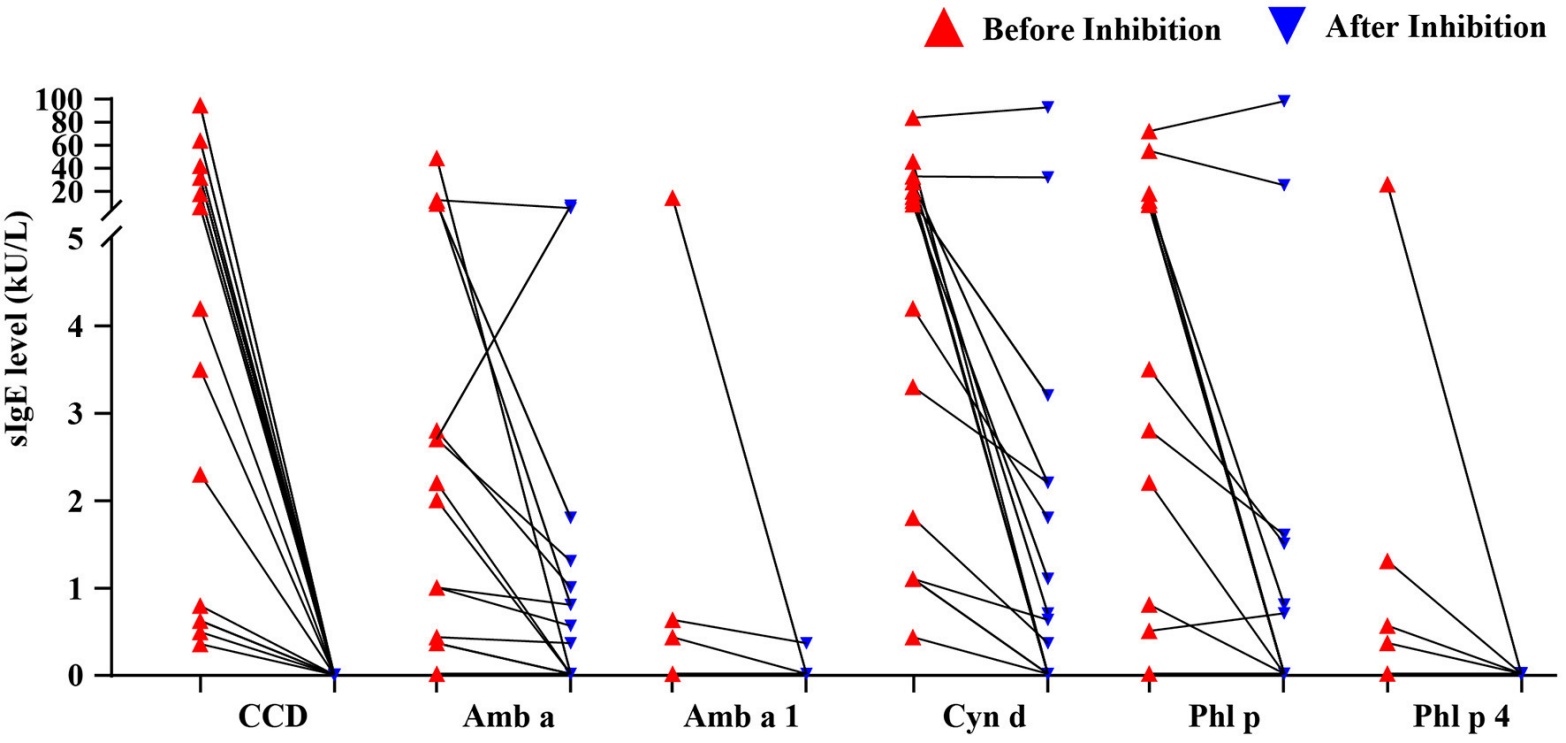
The sIgE levels of major grass pollen allergens before and after CCD inhibition.

The CCD inhibition test was carried out on the pollen allergens and components that were significantly positively correlated with the level of CCD-sIgE. The results show that the self-inhibition rate of CCD was more than 97%, almost completely. The median (first quartile, third quartile) inhibition rates of CCD on ragweed, Amb a 1, bermuda grass, timothy grass, and Phl p 4 sIgE were 72.4% (44.0, 97.2%), 97.7% (42.9, 99.9%), 89.7% (49.9, 99.1%), 96.0% (48.7, 99.9%) and 98.7% (97.%, 99.6%), respectively (Figure 4).

### Mugwort Cosensitization With Other Grass Pollen Allergens or Components in Different sIgE Levels

**Figure 5:**
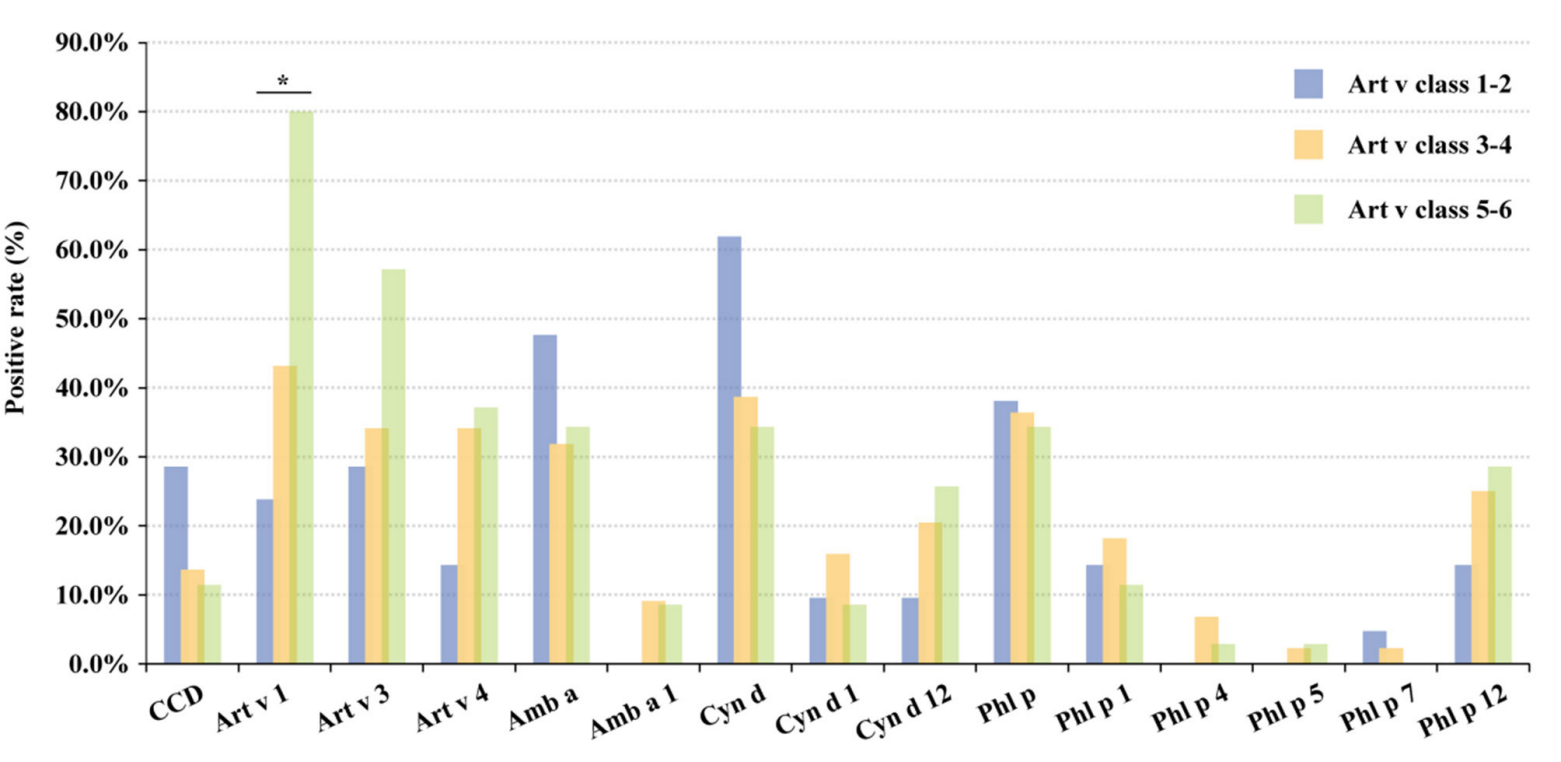
The positive rate of grass pollen allergens in different sIgE levels. The classifications are defined as follows: Class 0, <0.35 kU/L; Class 1, 0.35–0.70 kU/L; Class 2: 0.70–3.50 kU/L; Class 3: 3.50–17.50 kU/L; Class 4: 17.50–50.00 kU/L; Class 5: 50.00–100.00 kU/L and Class 6: ≥100.00 kU/L. *, *P* < 0.05. CCD, cross-reactive carbohydrate determinants; CRD, component-resolved diagnosis; sIgE, specific IgE; AIR-SKLRD, Allergy Information Repository of State Key Laboratory of Respiratory Disease; Art v, Artemisia vulgaris; Amb a, Ambrosia artemisiifolia; Cyn d, Cynodon dactylon; Phl p, Phleum pratense; AIC, Akaike Information Criterion; BIC, Bayesian Information Criterion; *r*_s_, Spearman's correlation coefficient.

Among 100 mugwort sIgE-positive patients, 21.0, 44.0, and 35.0% showed mild sensitization (Class 1–2), moderate sensitization (Class 3–4), and severe sensitization (Class 5–6), respectively. The higher the mugwort sIgE level, the higher the positive rates in Art v 1, Art v 3, Art v 4, Cyn d 12, and Phl p 12, and the lower in bermuda, timothy, and CCD. However, there was no significant difference in positive rates of other allergens among three different mugwort sIgE levels (mild, moderate, and severe sensitization) except Art v 1 (*P* < 0.05; Figure 5). In addition, the multi-allergen (2 or above) sensitization rates of patients with mild, moderate, and severe sensitization were 61.9, 56.8, and 88.6%, respectively (*P* = 0.007).

## Discussion

In the past 30 years, the incidence of allergy has increased significantly in China, and it is confirmed that allergies are a major risk factor for many diseases, for instance, asthma (22). Of note, mugwort was the main pollen species causing seasonal allergic asthma in China, and 11.3% of patients with respiratory allergic disease in China were allergic to mugwort (9, 23–25). In this study, we first disclosed that the patterns of molecular sensitization to mugwort pollen and its cosensitization with other grass pollens (ragweed, bermuda grass, and timothy grass) in mugwort-sensitized child allergy patients in western China. The major findings are summarized in the following four points. (1) Art v 1 was the main allergenic component of mugwort-sensitized children in western China; (2) Art v 4 was the typical cross-reaction component that was cosensitized to Phl p 12 and Cyn d 12; (3) CCD may have less interference in the detection of mugwort, and there was wide cross-reaction between ragweed, bermuda grass, and timothy grass caused by CCD; (4) the higher the sIgE level of mugwort in clinical detection, the more likely the child is to be sensitized to the real allergenic components of mugwort.

CRD is an advanced method for the diagnosis of allergens, which is helpful in the personalized management and treatment of allergic diseases. At present, the routine diagnosis of allergy to mugwort in China mainly depends on a skin prick test, ImmunoCAP, or immunoblotting kit. CRD is rarely used to explore the sensitization patterns or to guide allergen immunotherapy among Chinese patients with mugwort allergy. Additionally, there are currently only two allergen components (Art v 1 and Art v 3) of mugwort on the market that can be used for CRD. To fill this gap, the EUROIMMUN system, a new CRD method, was used to analyze the sIgE-positive patients of mugwort in this study. EuroLine (EUROIMMUN Medizinische Labordiagnostika AG, Lübeck, SH, Germany), as an IgE determination system, is based on the principle of the immunoblotting assay, which only needs 150 μL of serum. This method is mainly used for a semi-quantitative *in vitro* assay of allergen-specific IgE and has good diagnostic efficiency (26, 27).

Currently, a total of seven kinds of mugwort allergens are listed in the International Union of Immunological Societies (IUIS) database (28). An early study in Europe has disclosed that 60%−80% of patients could recognize Art v 1, Art v 3, Art v 4, or Art v 6, and the cosensitization rate of more than three mugwort pollen allergens was up to 60% (29). Moreover, previous studies report that about 80%−95% of patients with mugwort allergy were Art v 1–positive (30–32), 35–85% were Art v 3-positive (33, 34), 36% were Art v 4-positive (35). It is confirmed that Art v 1 and Art v 3 were the main allergen components of mugwort, which can be used as specific markers of allergy of mugwort (33, 36, 37). In comparison with others, our findings suggest that the positive rate of mugwort components Art v 1 among mugwort-sensitized patients was obviously lower (52.0%) although the positive rate of Art v 3 (41.0%) and Art v 4 (31.0%) were similar with others. Additionally, the results also reveal that the higher degree of sensitization is associated with the higher positive rate of Art v 1. Therefore, we speculated that the lower positive rate of Art v 1 in ours may be attributed to the relatively low level of sIgE of mugwort.

Our study reported that mugwort-sensitized patients were also more probably positive for ragweed, bermuda, timothy, and CCD sIgE. We found that 13.9% of patients with ragweed allergen sensitivities were positive for Amb a 1, which is the true allergenic component of ragweed allergen. Besides this, those patients were also positive for Art v 3. However, previous studies show that more than 95% of ragweed-allergic patients have Amb a 1-IgE (29), and the positive reaction of ragweed sIgE in this study may be related to the cross-reaction of mugwort. Meanwhile, 21.4% of patients who are bermuda grass allergen-positive were positive for Cyn d 1, the true allergen component of bermuda grass, and 38.1% were positive for Cyn d 12. All patients with Cyn d 12-sIgE were concomitant with Art v 4- sIgE and Phl p 12- sIgE, and there was a high correlation between Cyn d 12, Art v 4, and Phl p 12 (*r*_s_ > 0.75, *P* < 0.01). The results of LCA also supported the cosensitization of Cyn d 12, Art v 4, and Phl p 12. To our knowledge, we are the first to disclose that Art v 4 is the typical cross-reaction component of mugwort. The potential biological mechanism in Phl p 12, Art v 4, and Cyn d 12 belong to the prefibrin family, which has a highly conserved amino acid sequence. About 80% of the amino acid sequence of the prefibrin of plants from different sources is the same (38), and it can induce the IgE cross-reaction. In addition, 27.8 and 5.6% of patients who were positive for timothy allergens were positive for timothy components Phl p 1 and Phl p 5 as well as 44.4, 11.1, and 5.6% being positive for Phl p 12, Phl p 4, and Phl p 7, respectively. A similar study performed in Europe shows that Phl p 1 and Phl p 5 were the true allergenic components, and Phl p 4, Phl p 7, and Phl p 12 were cross-reactive components. Moreover, another study reports that the positive rate of Phl p 1 in patients with an allergy to timothy is 90–92% (39, 40). In comparison with others, the positive rate of Phl p 1 in this study is very low, and the positive rates of Phl p 12 and Phlp 4 are relatively high, which suggests the sensitization reaction to timothy is mainly caused by cross-reactive components.

We further explore the positive rate of grass pollen allergens in patients with different sensitization levels of mugwort. The results show that the higher sIgE of mugwort is associated with the higher sIgE positive rate of Art v 1, Art v 3, Art v 4, Cyn d 12, and Phl p 12 while being associated with lower bermuda grass, timothy grass and CCD. Although the difference among three various classes of sIgE did not achieve significance except Art v 1, it still implies that the sIgE level of mugwort is associated with the risks of being allergic to mugwort to some extent. Moreover, our study reports a high proportion of mugwort-sensitized patients with multiple allergens, which should be paid much attention because of its potential clinical implications.

Additionally, our research shows that the positive rate of CCD in mugwort-sensitized patients was 16%, which was larger than the 9% reported by Ebo et al. (41). The inconsistency of the CCD test results may be due to the differences in regions or detection methods. The Spearman correlation analysis reports that CCD has a strong positive correlation with ragweed, Amb a 1, bermuda grass, timothy, and Phl p 4, and the CCD inhibition test further confirms that there was a strong cross-reaction between CCD and these allergens. In clinical practice, much attention should be paid to the correct identification of the false positive of pollen allergen caused by CCD. To reduce the false positive interference, it is highly necessary to react with anti-CCD antibody adsorbent first and then detect the allergen sIgE. The results suggest that the correlation between CCD and mugwort or its components in this study is poor. The possible reason may be that the role of CCD-sIgE is quite limited in mugwort-sensitized children in western China. Similarly, a recent study by Gao et al. (30) also shows that recombinant Art v 1 has a similar IgE binding ability in mugwort allergy patients, which suggests CCD-sIgE has little effect on Art v 1 binding.

Several limitations also exist in this study. We did not investigate the concrete geographical distribution and living environment of these patients or collect information on the course and severity of the disease. Nevertheless, to our knowledge, this study may be the first to use CRD on sensitization to detect grass pollen allergens in China children. Additionally, a CCD inhibition test was also performed in this study. In the United States and Europe, multiallergen sensitization is more common than single allergen sensitization in patients with moderate-to-severe respiratory diseases (42). A study in the Philippines shows that the multisensitization rate of inhaled allergens in patients with allergic rhinitis can even reach 97.4% (43). Because of the lack of relevant clinical data, our study did not analyze multiallergen sensitization rates and disease severity. Future studies are highly expected to continue this topic with efforts that circumvent the limitations in our study.

In conclusion, mugwort sIgE-positive patients were co-sensitized with ragweed, bermuda grass, timothy grass, and CCD sIgE-positive to varying degrees. Among mugwort sIgE-positive patients, Art v 1 has the highest positive rate; moreover, the level of mugwort sIgE was positively associated with the positive rate of Art v 1. The cross-reaction components of mugwort with other pollen allergens are mainly caused by Art v 4. Additionally, the correlation between mugwort and CCD is poor, suggesting that CCD has little interference in the detection of mugwort sIgE. However, ragweed, Bermuda, and timothy grass allergens have a wide range of cross-reactions caused by CCD.

## Data Availability Statement

The original contributions presented in the study are included in the article/Supplementary Material, further inquiries can be directed to the corresponding author/s.

## Ethics Statement

This study involving human participants was reviewed and approved by the Medical Ethics Committee of the First Affiliated Hospital of Guangzhou Medical University (GYFYY-2018-93), and all experiments were conducted following the relevant guidelines and regulations of the Ethics Committee. The patients/participants [legal guardian] provided written informed consent to participant in this study.

## Author Contributions

CL, XH, BS, and XZ designed the study. LW and WL completed the data management and data cleaning as well as interpretation. XH and XZ performed the statistical analysis. CL and XH drafted the manuscript. CL, BS, and XZ edited the manuscript. CL, XH, HZ, XS, and YY contributed to the revision of this manuscript. All authors contributed to the article and approved the final version.

## Funding

This study was supported by the University of Macau (Grant Nos. FHS-CRDA-029-002-2017 and MYRG2018-00071-FHS) as well as the Science and Technology Development Fund, Macau SAR (File No. 0004/2019/AFJ and 0011/2019/AKP). This study was funded by the National Natural Science Foundation of China (Project No. 81871736, 81802076), Guangzhou Municipal Health Foundation (20191A011073), the Medicine and Health Care Technology Projects of Guangzhou (Project No. 2017A013010017), Guangzhou Science and Technology Foundation (Project Nos. 201804020043 and 202102010327), National Key Technology R&D Program (2018YFC1311900), Guangdong Science and Technology Foundation (2019B030316028), the Foundation of SKLRD (MS-2019-06 and Z-2022-09), and the Foundation of GYYY (ZH201904), ZNSA-2020012 and ZNSXS-20220013. The funders had no roles in study design, data collection, data analysis, interpretation and writing of the report.

## Conflict of Interest

The authors declare that the research was conducted in the absence of any commercial or financial relationships that could be construed as a potential conflict of interest.

## Publisher's Note

All claims expressed in this article are solely those of the authors and do not necessarily represent those of their affiliated organizations, or those of the publisher, the editors and the reviewers. Any product that may be evaluated in this article, or claim that may be made by its manufacturer, is not guaranteed or endorsed by the publisher.
