# Supplementary material for: Major Grass Pollen Allergen Components and Cross-Reactive Carbohydrate Determinants in Mugwort-Sensitized Child Patients With Allergic Respiratory Disease in Western China
Source: Front Pediatr. 2022 Apr 14;10:816354. doi: 10.3389/fped.2022.816354 (PMC9047046; doi:10.3389/fped.2022.816354)
Supplement: Supplementary file 1 [file Table_1.DOCX]

**Supplemental Table1** Model fit statistics of latent class analysis

| K-class | AIC | BIC | Entropy |
| --- | --- | --- | --- |
| 2 | 505.53 | 586.29 | 0.89 |
| 3 | 440.31 | 562.76 | 0.95 |
| 4 | 421.86 | 585.99 | 0.96 |
